# Kinematics but not kinetics alterations to single-leg drop jump movements following a subject-tailored fatiguing protocol suggest an increased risk of ACL injury

**DOI:** 10.3389/fspor.2024.1418598

**Published:** 2024-05-20

**Authors:** Mauro Nardon, Umberto Ferri, Giovanni Caffi, Manuela Bartesaghi, Cecilia Perin, Antonio Zaza, Cristiano Alessandro

**Affiliations:** ^1^ School of Medicine and Surgery/Sport and Exercise Medicine, University of Milano-Bicocca, Milan, Italy; ^2^ School of Medicine and Surgery/Physical and Rehabilitative Medicine, University of Milano-Bicocca, Milan, Italy; ^3^ Istituti Clinici Zucchi - GDS, Carate Brianza, Monza e Brianza, Italy; ^4^ Department of Biotechnology and Biosciences/Sport and Exercise Medicine, University of Milano-Bicocca, Milan, Italy

**Keywords:** anterior cruciate ligament, risk of injury, neuromuscular fatigue, landing, drop jump, sports biomechanics, kinematics, kinetics

## Abstract

**Introduction:**

Neuromuscular fatigue causes a transient reduction of muscle force, and alters the mechanisms of motor control. Whether these alterations increase the risk of anterior cruciate ligament (ACL) injury is still debated. Here we compare the biomechanics of single-leg drop jumps before and after the execution of a fatiguing exercise, evaluating whether this exercise causes biomechanical alterations typically associated with an increased risk of ACL lesion. The intensity of the fatiguing protocol was tailored to the aerobic capacity of each participant, minimizing potential differential effects due to inter-individual variability in fitness.

**Methods:**

Twenty-four healthy male volunteers performed single leg drop jumps, before and after a single-set fatiguing session on a cycle ergometer until exhaustion (cadence: 65–70 revolutions per minute). For each participant, the intensity of the fatiguing exercise was set to 110% of the power achieved at their anaerobic threshold, previously identified by means of a cardiopulmonary exercise test. Joint angles and moments, as well as ground reaction forces (GRF) before and after the fatiguing exercise were compared for both the dominant and the non-dominant leg.

**Results:**

Following the fatiguing exercise, the hip joint was more extended (landing: Δ=−2.17°, *p* = 0.005; propulsion: Δ=−1.83°, *p* = 0.032) and more abducted (landing: Δ=−0.72°, *p* = 0.01; propulsion: Δ=−1.12°, *p* = 0.009). Similarly, the knee joint was more extended at landing (non-dominant leg: Δ=−2.67°, *p* < 0.001; dominant: Δ=−1.4°, *p* = 0.023), and more abducted at propulsion (both legs: Δ=−0.99°, *p* < 0.001) and stabilization (both legs: Δ=−1.71°, *p* < 0.001) hence increasing knee valgus. Fatigue also caused a significant reduction of vertical GRF upon landing (Δ=−0.21 N/kg, *p* = 0.003), but not during propulsion. Fatigue did not affect joint moments significantly.

**Conclusion:**

The increased hip and knee extension, as well as the increased knee abduction we observed after the execution of the fatiguing exercise have been previously identified as risk factors for ACL injury. These results therefore suggest an increased risk of ACL injury after the execution of the participant-tailored fatiguing protocol proposed here. However, the reduced vertical GRF upon landing and the preservation of joint moments are intriguing, as they may suggest the adoption of protective strategies in the fatigued condition to be evaluated in future studied.

## 1 Introduction

Neuromuscular fatigue (NMF) is a physiological phenomenon defined as a transient reduction in the capability of a muscle or muscle group to generate force or power following physical exercise (1–3). The state of fatigue often results in performance degradation, observed during the execution of several motor tasks including balance (4), walking (5–8), reaching movements (9), dynamic loading during running (10, 11), and shock absorption during landing (12, 13). Furthermore, recent results suggests that neuromuscular fatigue also affects basic mechanisms of human motor control, causing transient alterations to movement planning (14), sensorimotor integration (15, 16), joint position sense (17) and postural control (18–20). Whether these alterations in force generation and control mechanisms lead to inappropriate motor commands (21) that increase the risk of musculoskeletal injury is still unclear.

One of the most common sport-related injuries is the anterior cruciate ligament (ACL) tear (22). Although surgical reconstruction and rehabilitation, this lesion may lead to withdrawal from competitive sports (23), or to an increased risk of developing secondary knee pathologies, including ACL re-injuries and osteoarthritis (24, 25). Identifying the risk factors associated with this injury is of great importance to reduce the incidence of these events. In addition to unmodifiable subjective characteristics that may predispose athletes to this injury (i.e., anthropometric features and gender) (26, 27), specific movement biomechanics are often associated to ACL tears (28, 29). These aberrant biomechanics typically involve increased knee abduction angles (29, 30) and external knee abduction moments (29), increased ground reaction forces (29), as well as reduced hip and knee flexion angles at landing (31, 32). For this reason, specific preventive training and rehabilitation after injury have been developed to improve control over potentially hazardous movements (33). Nonetheless, it is still possible that alterations in motor control due to fatigue may facilitate these aberrant movement biomechanics, a notion likely relevant to the design of training programs.

Whether neuromuscular fatigue constitutes a risk factor for ACL injury is however still controversial (34). Recent literature has shown inconsistent results on this issue, with some studies suggesting that fatigue is associated with increased risk of ACL injuries (35, 36) and other suggesting no association (34, 37). These contrasting results may be due to the heterogeneity of the protocols used to induce fatigue across studies (38, 39). In addition, these protocols typically require the execution of exercises until exhaustion either without controlling exercise intensity (35, 40–42) or without adapting exercise intensity to the fitness level of each participant (13, 37, 43). Since the physiological responses to physical exercise largely depend on exercise intensity relative to individual ventilatory thresholds (44), this approach may induce differential effects across participants with different fitness levels (43). Therefore, these protocols may lead to incoherent fatigue-induced biomechanical alterations, confounding the obtained results (34).

**Figure 1:**
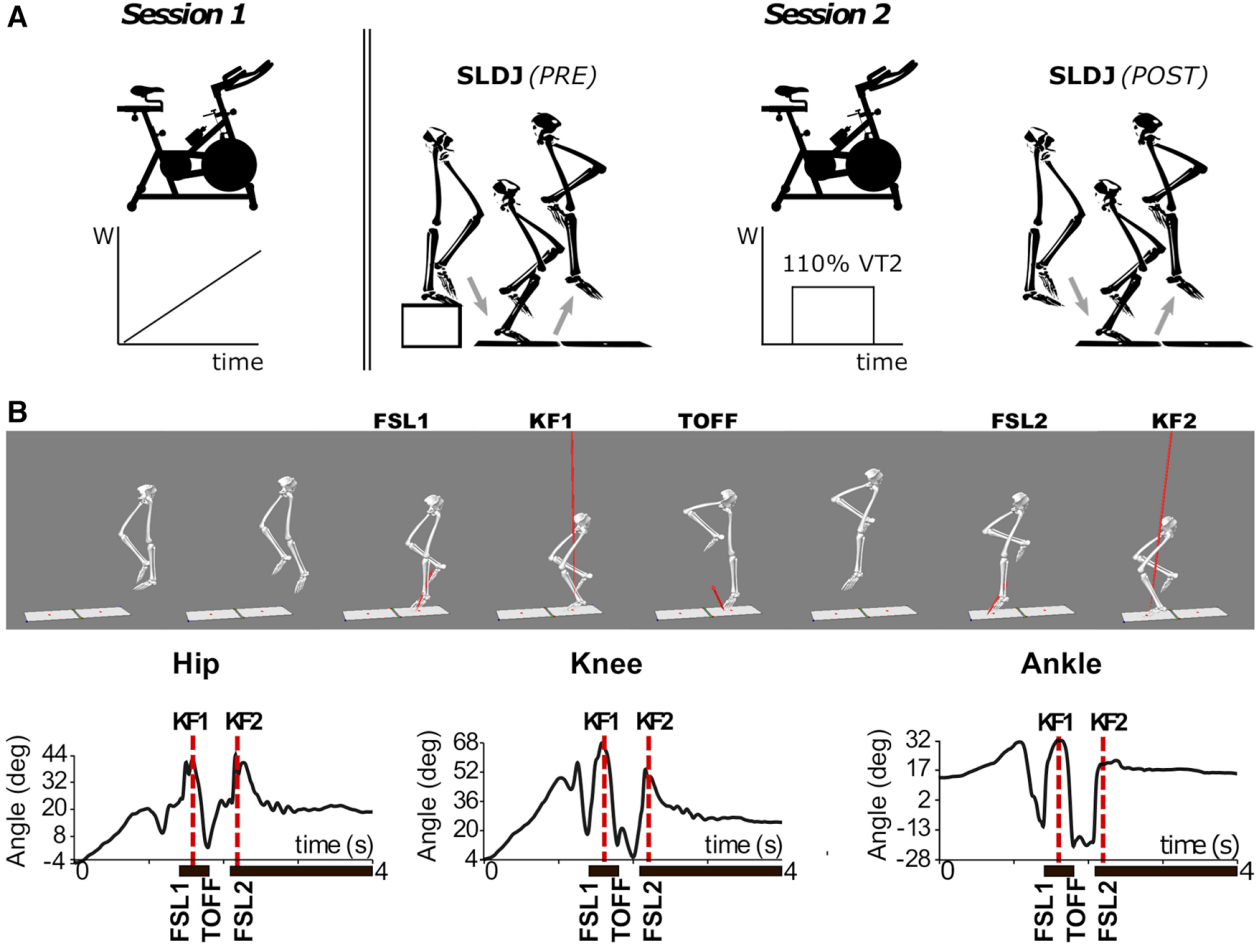
(**A**) Schematic outline of the experimental protocol. Session 1: incremental CPET on a cycle-ergometer. Session 2: single-leg drop jump (SLDJ) before (PRE) and after (POST) the subject-tailored fatiguing protocol on the cycle-ergometer. (**B**) 3D reconstruction (upper panel) and sagittal plane joint angles (lower panel) during the execution of a SLDJ movement for a representative subject. Note that the events of peak knee flexion (KF1 and KF2) were identified solely from the kinematics of the knee, but are closely related to the timepoints corresponding to hip and ankle joint angles peaks. FSL1, timepoint of foot strike at landing 1; FSL2, timepoint of foot strike at landing 2; KF1, timepoint of peak knee flexion during landing 1; KF2, timepoint of peak knee flexion during landing 2; SLDJ, single-leg drop jump; VT2, second ventilatory threshold; W, power output (Watt).

To overcome this problem, here we evaluate alterations in movement biomechanics following a standardized fatiguing protocol adapted to the fitness level of each participant (Figure 1A). We compare the biomechanics of single-leg drop jump movements before and after a fatiguing session on a cycle-ergometer until exhaustion. The exercise protocol was individualized to participants’ fitness level by setting the workload to 110% the power associated to the anaerobic ventilatory threshold (VT2) of each participant, previously identified by means of a standardized cycle-ergometer cardiopulmonary exercise test (CPET). We specifically designed the fatiguing protocol to induce a high level of physiological stress at both the cardiopulmonary and metabolic systems, caused by both the accumulation of metabolites (44) and the loss in muscle efficiency (45). This is arguably a more similar exercise to that performed during collective sport, than exercise protocols that target localized muscle groups (41, 43). We then evaluate if this generalized fatiguing protocol causes movement alterations typically associated with an increased risk of ACL injury, such as increased knee valgus and abduction moments as well as increased knee extension (29).

## 2 Material and methods

### 2.1 Participants

Twenty-four healthy male volunteers with no history of musculoskeletal, cognitive, cardiopulmonary, nor neurological disorders participated in this study after signing and informed consent (age: 29.3 ± 4.2 years; weight: 76.1 ± 8.0; height: 179.9 ± 6.3; BMI: 23.5 ± 1.8). Subjects did not practice sport at competitive level, and had different sport backgrounds: skiing, ice-skating, swimming, soccer, martial arts, running, bodybuilding, weightlifting, water polo, cycling. All the procedures were approved by the local ethics committee of the University of Milano-Bicocca and were conducted in conformance with the Declaration of Helsinki.

### 2.2 Study protocol

Participants took part in two experimental sessions, separated by at least 48 h. During the first session (*Session 1*), they performed a cardiopulmonary exercise test (CPET) on a cycle-ergometer (Monark LC6, COSMED). The test followed a standardized incremental ramp protocol: after 1 min of rest and two minutes of warm up cycling against no resistance, the workload of the cycle-ergometer increased constantly by 30 W/min; participants were asked to maintain a cadence of 65–70 repetitions per minute until exhaustion, with verbal encouragement. An experienced specialist in Sport Medicine (author MB) supervised all the tests and performed offline analyses to identify the ventilatory thresholds using the Wasserman method (46–48). The workload associated to the 2^nd^ ventilatory threshold (VT2—or anaerobic threshold) was used during the second experimental session to tailor the intensity of the fatiguing protocol to each participant's fitness level.

During the second session (*Session 2*), participants performed a battery of functional tests before (*PRE*) and after (*POST*) a fatiguing exercise on the cycle-ergometer (Figure 1A). These tests were: single-leg drop jump (SLDJ), single-leg hop for distance, stabilometry and cutting maneuvers, executed in random order across participants. This work focuses on the SLDJ: participants were instructed to drop from a step (height: 21 cm) onto a force platform with one foot, and perform a vertical jump as quick and high as possible, landing and finally stabilizing with the same foot on a second force platform (Figures 1A,B). Repetitions were considered invalid if the foot was not entirely on the force platform, or when subjects could not maintain balance upon landing. At least four valid repetitions were recorded for each subject and leg. The dominant leg of each participant was identified by asking what leg participants would use to kick a ball (right leg for all the subjects) (49).

The fatiguing exercise consisted in a single bout of cycling at a constant workload, maintaining a cadence of 65–70 rpm with verbal encouragement until exhaustion: after 10 min of warm-up (5 min at 70 W followed by 5 min at 50% of the VT2 workload), the ergometer load was set to 110% of the individual VT2 workload, thus effectively obtaining an anaerobic fatiguing exercise with intensity tailored to the individual fitness level. Upon exercise termination, participants reported their perceived exertion on a Borg scale [6–20 (50);] as a measure of internal load. After the fatiguing exercise, when the participant's heart rate and respiratory frequency (as monitored by portable devices, see Data acquisition) stabilized to pre-exercise levels to avoid movement artefacts due to hyperventilation, the functional tests were repeated in the same order as before the fatiguing exercise.

### 2.3 Data acquisition

During Session 1, breath-by-breath cardiopulmonary parameters were recorded continuously using a portable metabolimeter (K5, COSMED) and a 12-derivations electrocardiograph (Quark C12x-T12x, COSMED). These data were integrated, displayed and saved using a dedicated software (Omnia, COSMED), which also controlled the resistance of the cycle-ergometer and therefore the exercise workload.

During Session 2, retroreflective passive markers (diameter: 2 cm) were placed bilaterally on anatomical landmarks on the pelvis and the lower limbs: posterior-superior iliac spine, antero-superior iliac spine, iliac crest, greater trochanter, lateral femoral condyle, caput fibulae, tibial tuberosity, lateral malleolus, calcaneus, distal part of V° feet metatarsal bone and instep of the foot, medial femoral condyle, medial malleolus and distal part of I° feet metatarsal bone. Additionally, two clusters were positioned on the thigh and shank segments of each leg. The 3D motion capture system (179 Hz) consisted of 8 infra-red cameras (Oqus 500+, Qualisys) and two video cameras (Oqus 210c, Qualisys), and was synchronized with two force platforms (size: 46 × 51 cm; model: OR6-7, AMTI, Watertown, MA, US; 2,148 Hz). During the fatiguing cycling exercise, participants cardiopulmonary parameters were monitored using a portable metabolimeter (K5, COSMED) and an HR monitor (HRM-Dual™, Garmin Inc) integrated and displayed by a dedicated software (OMNIA, COSMED).

### 2.4 Data processing and features

Markers trajectories were visually inspected and labelled using the software Qualisys Track Manager. Gaps shorter than 5 frames were filled using linear interpolation. Data were then exported into Visual3D (version 2020.11.2, HAS-Motion) for further analyses. Markers trajectories and ground reaction forces (GRF) were low-pass filtered at the same cut-off frequency (4^th^ order Butterworth, 15 Hz) (51). Joint angles and moments were then computed in the sagittal (x), frontal (y), and transverse (z; only for knee and hip angles) planes. Joint moments and GRF were normalized by bodyweight to allow across-subjects comparisons.

Joint angles were evaluated at the following events (Figures 1B top): Foot strike at the 1^st^ landing (FSL1; the timepoint when the vertical component of the GRF—vGRF—exceeds 50 N); Peak knee flexion during the 1^st^ landing (KF1; the timepoint corresponding to the maximum value of knee flexion between FSL1 and the moment the foot leaves the ground, i.e., toe-off); Foot strike at the 2^nd^ landing (FSL2; identified like FSL1); and Peak knee flexion during the 2^nd^ landing (KF2).

**Figure 2:**
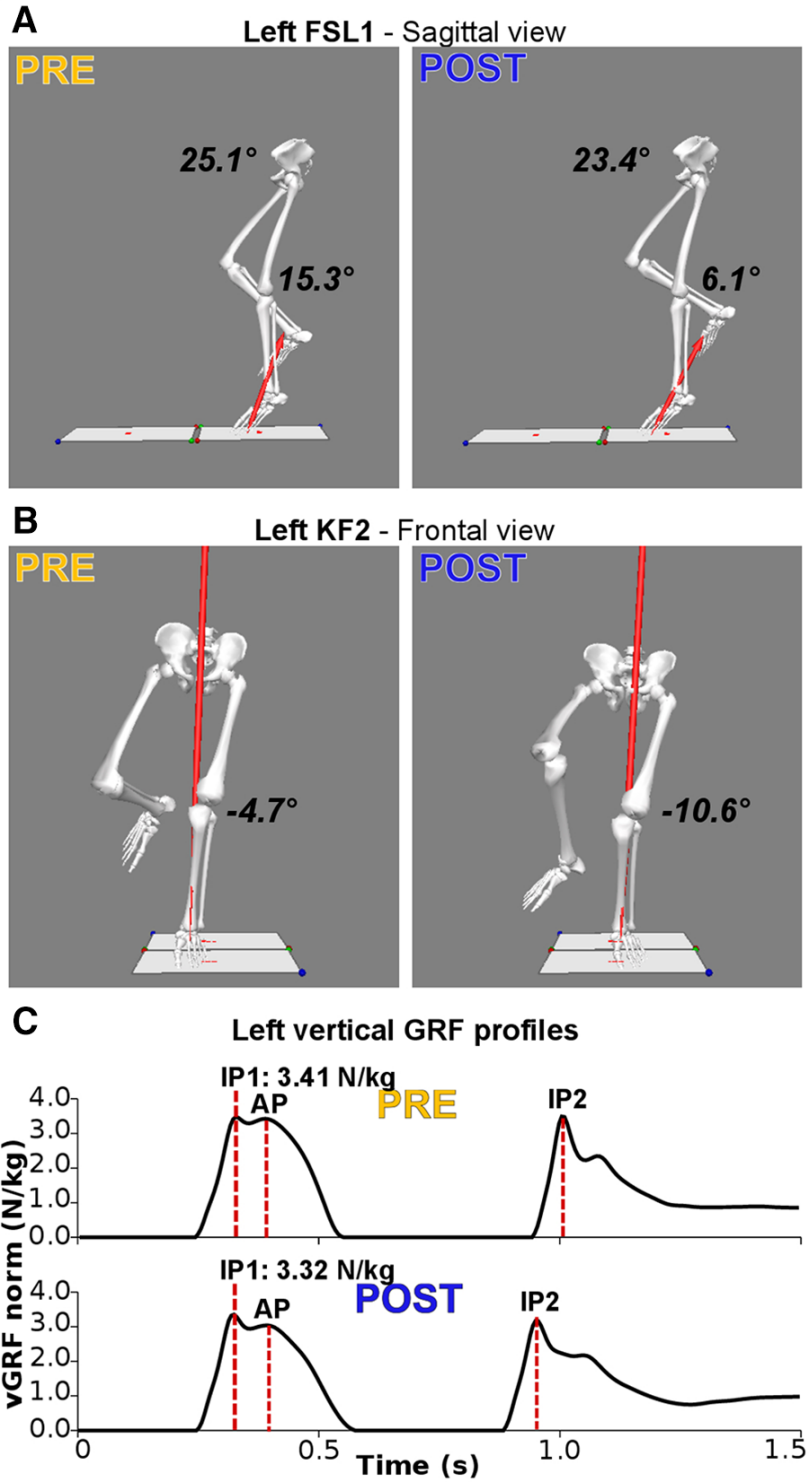
Main significant alterations following the fatiguing protocol for a representative subject. (**A**) Three-dimensional reconstruction of the lower limbs at foot-strike of the first landing (FSL1) in the sagittal plane. Following the fatiguing protocol (POST), hip (23.4° vs. 25.1°) and knee (6.1° vs. 15.3°) joints are more extended. (**B**) Three-dimensional reconstruction of the lower limbs at the instant of peak knee flexion (KF1) in the frontal plane. Following the fatiguing protocol, the knee is more abducted (−10.6° vs. −4.7°; i.e., increased knee valgus). (**C**) Bodyweight-normalized vertical Ground Reaction Force (vGRF) during SLDJ before (PRE) and after the fatiguing exercise (POST), with indications of the events of impact peaks (IP) and active peaks (AP). Following fatigue there is a reduction of the vGRF impact peaks during the first (IP1; PRE: 3.41 *N*/kg; POST: 3.32 *N*/kg).

Normalized vGRF was evaluated at the following events (Figure 1B bottom): Impact Peaks of the two landings (IP1 and IP2; the first local maxima in the vGRF profiles after FSL1 and FSL2 respectively); and the Active Peak (AP; the 2^nd^ peak in the vGRF profile after FSL1, corresponding to the propulsive phase of the jump). Similarly, normalized joint moments were evaluated at IP1 and at the timepoint of their peak values (Figure 2).

As an index of functional performance, we computed jump height as the highest vertical position of the pelvis frame of reference during the time interval between toe-off and FSL2. To allow cross-subjects comparisons, this value was normalized by subtracting the pelvis height during a static standing pose in natural position. Finally, we computed contact time (CT) as the time elapsed between FSL1 and T_OFF_.

### 2.5 Statistics

We evaluated the effect of fatigue on repetition-averaged kinematic, kinetic, and functional variables (see above) for the two legs. For each variable under investigation, we conducted a repeated-measure two-ways analysis of variance (ANOVA) with factors *Fatigue* (Pre vs. Post—repeated measures), *Leg* (Dominant vs. Non-dominant; D-vs.-ND) and their interaction. When the interaction term was statistically significant, post-hoc tests with Bonferroni's correction were conducted using repeated measures t-tests, comparing the values of the dependent variables pre and post fatigue for each leg. Statistical analyses, including evaluating the assumptions of the statistical tests, were performed using IBM SPSS Statistics (version 29; IBM Corp). We considered differences to be statistically significant if the *p*-value for the null-hypothesis was <0.05. Data in text, tables, and figures—unless otherwise stated—are presented as mean ± standard deviation (s.d.). Out of the 24 recruited subjects, one participant did not complete the post-fatigue assessment due to minor muscular injury following the cycling exercise. For another participant, joint kinematics in the dominant leg could not be computed due to technical issues, resulting in a sample size of 22 and 23 participants for the analyses on the dominant and non-dominant leg respectively.

## 3 Results

During the CPET performed at Session 1, participants’ peak oxygen consumption (V˙O_2_ peak) was 43.3 ± 6.8 ml/kg/min, which falls within normative values for active male subjects in their age range (52). The average exercise workload corresponding to VT2 was 205 ± 39 W. During the fatiguing exercise (Session 2), participants reached exhaustion in an average of 6:57 ± 6:32 min:sec from the installment of the target subject-tailored workload. Upon termination, they reported a value of perceived exertion of 19.3 ± 0.6 on the Borg scale, thus confirming the fatiguing effect of the exercise. The effects on motor performance and movement biomechanics were as follows.

### 3.1 Motor performance in the SLDJ

Jump height was significantly influenced by factors *Fatigue* (*p* = 0.025) and *Leg* (*p* = 0.009), with a significant interaction term (*p* = 0.021). The post-hoc tests revealed a statistically significant reduction of jump height [Δ=−1.8 cm; 95% CI: (−2.6, −0.9); i.e., −7.8% of pre-fatigue jump height] only for the non-dominant leg (D: p = 0.735; ND: p = 0.006). There were no significant changes in contact time following fatigue (*p* = 0.751) nor across legs: *Leg* (*p* = 0.113).

### 3.2 Movement kinematics during the SLDJ

#### 3.2.1 Hip joint angles

**Figure 3:**
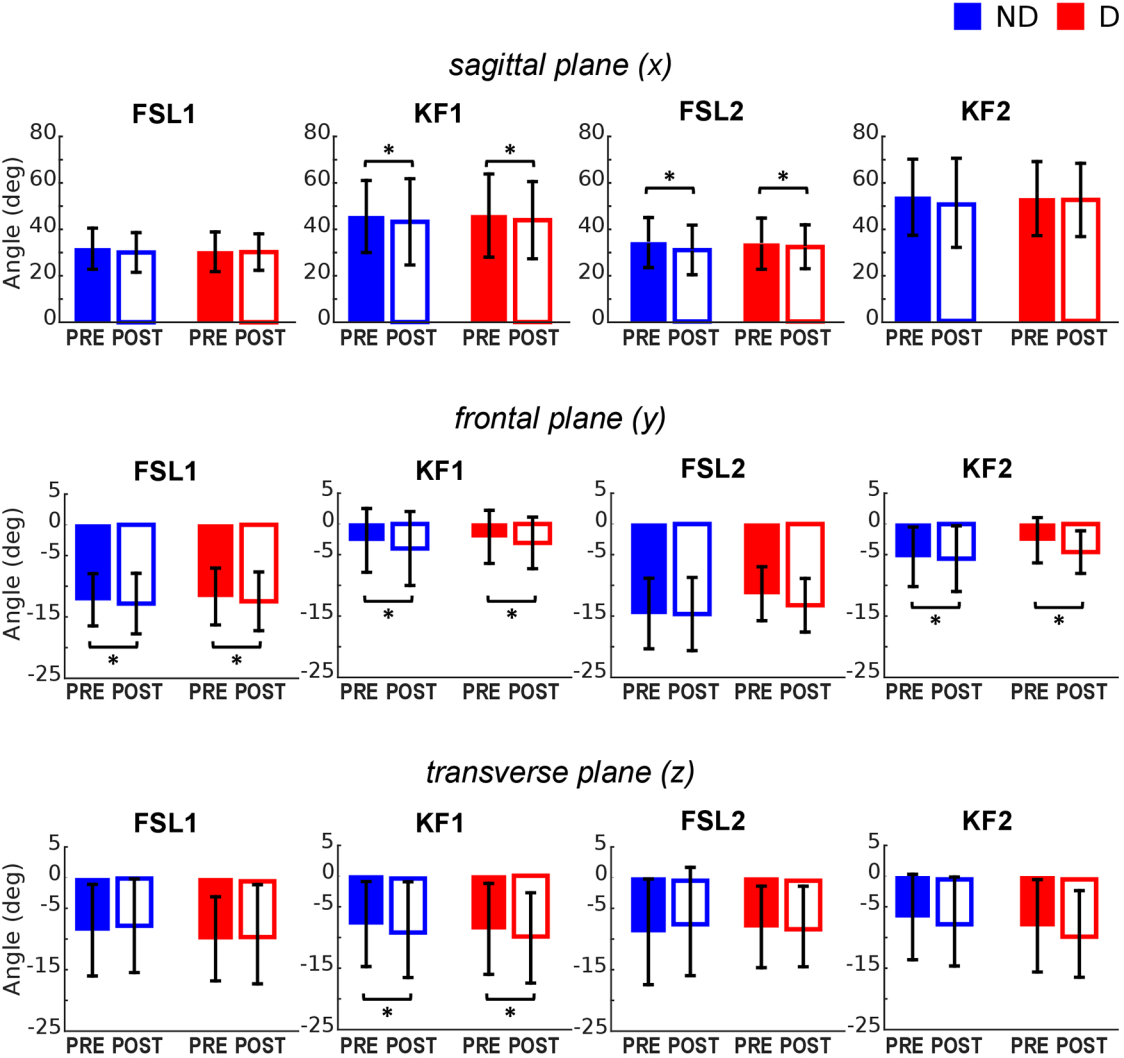
Hip joint angles pre and post the execution of the fatiguing protocol in the three anatomical planes. The execution of the fatiguing exercise caused: in the sagittal plane, a reduction of hip flexion at FSL1 for the non-dominant leg (**D**) and at KF1 and KF2 for both dominant (**D**) and non-dominant (ND) legs after the execution of the fatiguing protocol for both legs; in the frontal plane, an increase in hip abduction at FSL1, KF1 and KF2; in the transverse plane, an increase of hip extra-rotation at KF1 for both legs. Bar-plots represent mean ± standard deviation (s.d.; *N* = 23); significant differences are marked with asterisks (*). FSL1, foot strike at landing 1; FSL2, foot strike at landing 2, KF1, time of knee peak flexion during landing 1; KF2, time of knee peak flexion during landing 2.

The ANOVA (Table 1) indicated a significant reduction of hip flexion at KF1 and FSL2 following the fatiguing exercise for both legs in the sagittal plane (Figures 2A, 3 top). In the frontal plane (Figure 3 middle), there was a significant reduction of hip joint angle at FSL1, KF1 and FSL2 following the fatiguing exercise independently of leg, indicating that for both legs the femur was more abducted relative to pelvis. In the transverse plane (Figure 3 bottom), we found a significant difference of hip angle at KF1 due to fatigue independently of leg, with the femur more externally rotated for both legs following the fatiguing exercise. Results of the statistical analyses are reported in Table 1.

**Table 1:** Statistical analyses for the hip joint angles.

Plane	Event	Fatigue	Leg	Fatigue*Leg	Post-hoc		Δ (95% CI)
					ND	D	
*Sagittal*	FSL1	.120	.527	.092	** **	** **	** **
	KF1	.**032**	.522	.863	** **	** **	−1.83 (−3.48, −0.18)
	FSL2	.**005**	.477	.113	** **	** **	−2.17 (−3.6, −0.75)
	KF2	.122	.593	.146	** **	** **	** **
*Frontal*	FSL1	.**010**	.893	.870	** **	** **	−0.72 (−1.25, −0.19)
	KF1	.**009**	.608	.787	** **	** **	−1.12 (−1.93, −0.30)
	FSL2	.073	.098	.115	** **	** **	** **
	KF2	.**007**	.094	.167	** **	** **	−1.1 (−1.87, −0.34)
*Transverse*	FSL1	.155	.338	.993	** **	** **	** **
	KF1	.**027**	.385	.517	** **	** **	−1.03 (−1.93, −0.13)
	FSL2	.078	.984	.102	** **	** **	** **
	KF2	.069	.217	.518	** **	** **	** **

*P*-values associated to the factors *Fatigue* (Pre, Post), *Leg* (Dominant(D), Non-dominant (ND)) and their interactions resulting from the repeated measure ANOVA at different timepoints, as well as *p*-values associated to the post-hoc tests to evaluate potential differential effects of fatigue across legs. Post-hoc tests were performed if the Fatigue*Leg interaction was significant, reporting the mean differences (Δ) between the pre- and post-values of the joint angle, along with the corresponding 95% confidence intervals (CI), for each leg individually (D and ND). Otherwise, a single mean difference is reported, as the non-significance of the interaction term indicates that the factor *Fatigue* has the same effect on both legs. Significant *p*-values are highlighted in bold.

#### 3.2.2 Knee joint angles

**Figure 4:**
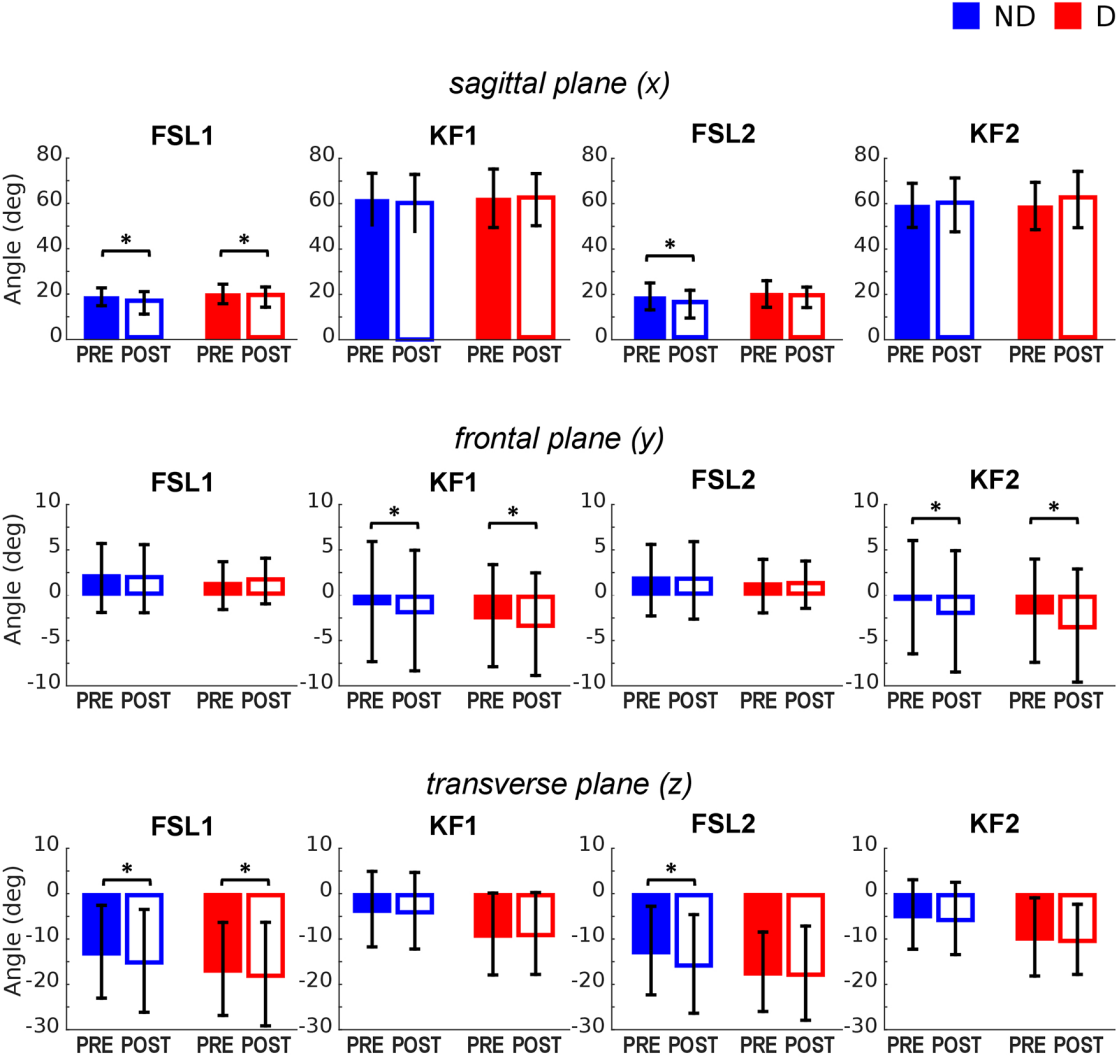
Knee joint angles pre and post the execution of the fatiguing protocol in the three anatomical planes. The execution of the fatiguing exercise caused: in the sagittal plane, a reduction of knee flexion at FSL1 in both legs, and at FLS2 in the ND leg; in the frontal plane, an increase of knee abduction at KF1 and KF2 in both legs; in the transverse plane, an increase in knee external rotation in both legs at FSL1, and in the ND leg at FSL2. Bar-plots represent mean ± standard deviation (s.d.; *N* = 23); significant differences are marked with asterisks (*). FSL1, foot strike at landing 1; FSL2, foot strike at landing 2, KF1, time of knee peak flexion during landing 1; KF2, time of knee peak flexion during landing 2.

The ANOVA (Table 2) indicated a significant effect of *Fatigue* and a significant Fatigue*Leg interaction at FSL1 and FSL2 in the sagittal plane, suggesting a reduction of knee flexion following fatigue. Post-hoc tests revealed that this reduction occurred for both legs at FSL1 and only for the ND leg at FSL2 (Figures 2A, 4 top). In the frontal plane, there was a significant effect of *Fatigue* at KF1 and KF2 independently of *Leg*, indicating an increase in knee abduction following the fatiguing protocol (i.e., an increase in knee valgus; Figures 2B, 4 middle). In the transverse plane, we found significant differences in knee angle at FSL1 for both legs and at FSL2 only for the ND leg following the fatiguing exercise, with an increase in the external rotation of the tibia relative to femur (Figure 4 bottom). Results of the statistical analyses are reported in Table 2.

**Table 2:** Statistical analyses for the knee joint angles.

Plane	Event	Fatigue	Leg	Fatigue*Leg	Post-hoc		Δ (95% CI)
					ND	D	
*Sagittal*	FSL1	**<.001**	.**023**	.057	**<**.**001**	.**023**	ND: −2.67 (−3.7, −1.62)
							D: −1.4 (−2.6, −0.21)
	KF1	.247	.221	.571	** **	** **	** **
	FSL2	**<.001**	.**002**	.046	**<**.**001**	.074	ND: −0.91 (−1.39, −0.41)
	KF2	.148	.354	.118	** **	** **	** **
*Frontal*	FSL1	.499	.384	.150	** **	** **	** **
	KF1	**<.001**	.218	.797	** **	** **	−0.997 (−1.53, −0.47)
	FSL2	.710	.888	.066	** **	** **	** **
	KF2	**<.001**	.187	.743	** **	** **	−1.712 (−2.37, −1.06)
*Transverse*	FSL1	.**003**	.111	.292	** **	** **	−1.63 (−2.63, −0.62)
	KF1	.504	.**003**	.344	** **	** **	** **
	FSL2	.**003**	.127	.**034**	.**002**	.720	ND: −2.93 (−4.61, −1.25)
	KF2	.182	.**003**	.598	** **	** **	** **

*P*-values associated to the factors *Fatigue* (Pre, Post), *Leg* (Dominant(D), Non-dominant (ND)) and their interactions resulting from the repeated measure ANOVA at different timepoints, as well as *p*-values associated to the post-hoc tests to evaluate potential differential effects of fatigue across legs. Post-hoc tests were performed if the Fatigue*Leg interaction was significant. Mean difference (Δ) between the pre- and post-values of the joint angle are reported, along with the corresponding 95% confidence intervals (CI). Significant *p*-values are highlighted in bold.

#### 3.2.3 Ankle joint angles

The ANOVA indicated no statistically significant differences in sagittal ankle angle due to *Fatigue*. In the frontal plane, there was a significant difference following fatigue at KF2, where the ankle was more adducted. Results of the statistical analyses are reported in Table 3.

**Table 3:** Statistical analyses for the ankle joint kinematics.

Plane	Event	Fatigue	Leg	Fatigue*Leg	Post-hoc		Δ (95% CI)
					ND	D	
*Sagittal*	FSL1	.161	.**025**	.613	** **	** **	** **
	KF1	.998	.541	.904	** **	** **	** **
	FSL2	.403	.**010**	.221	** **	** **	** **
	KF2	.331	.**034**	.176	** **	** **	** **
*Frontal*	FSL1	.427	.416	.220	** **	** **	** **
	KF1	.485	.169	.425	** **	** **	** **
	FSL2	.388	.737	.328	** **	** **	** **
	KF2	.**008**	.610	.445	** **	** **	1.57 (0.44, 2.68)

*P*-values associated to the factors *Fatigue* (Pre, Post), *Leg* (Dominant(D), Non-dominant (ND)) and their interactions resulting from the repeated measure ANOVA at different timepoints, as well as *p*-values associated to the post-hoc tests to evaluate potential differential effects of fatigue across legs. Post-hoc tests were performed if the Fatigue*Leg interaction was significant or close to significant. Mean differences (Δ) between the pre- and post-values of the joint angle are reported, along with the corresponding 95% confidence intervals (CI). Significant *p*-values are highlighted in bold.

### 3.3 Movement kinetics during the SLDJ

#### 3.3.1 Vertical ground reaction forces

**Figure 5:**
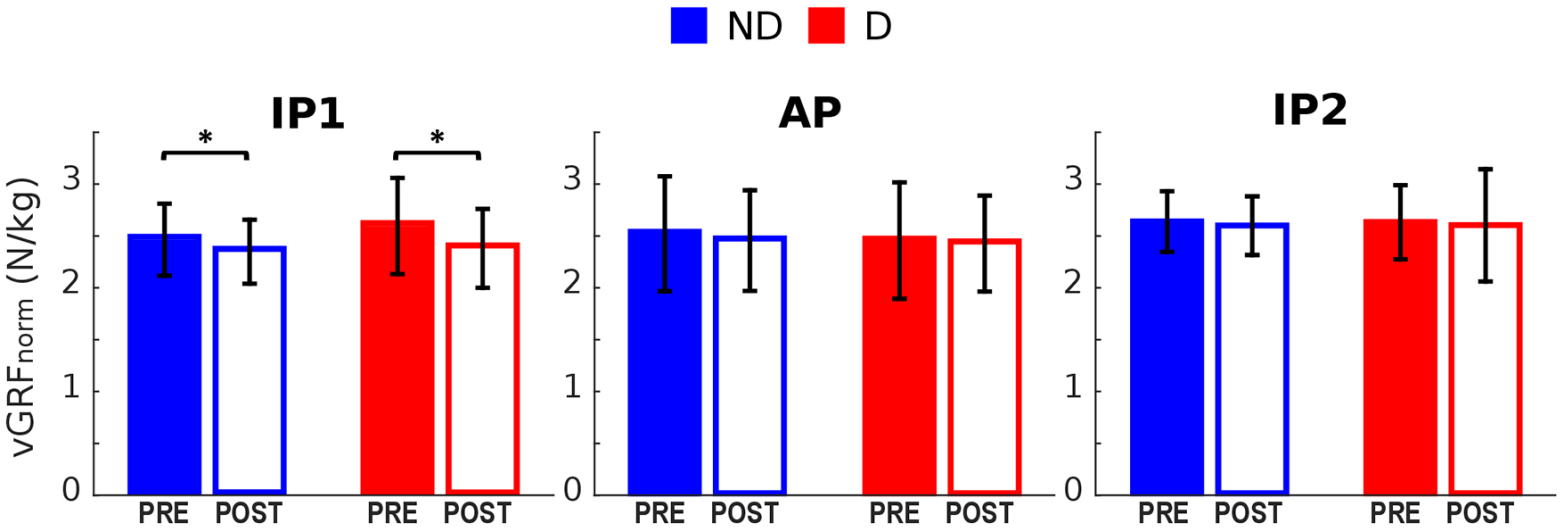
Vertical GRF pre and post the execution of the fatiguing protocol. The execution of the fatiguing exercise caused a reduction of the vGRF Impact Peak of the first landing for both dominant (**D**) and non-dominant (ND) legs. Bar-plots represent mean ± s.d (*N* = 23); significant differences are marked with asterisks (*). IP1, impact peak of the first landing; AP, active peak; IP2, impact peak of the second landing.

The vGRF peak at the first landing (IP1) was significantly reduced in both legs following the execution of the fatiguing exercise (Figures 2C, 5). No significant differences were found in vGRF at AP and IP2. Results of the statistical analyses are reported in Table 4.

**Table 4:** Statistical analyses for the vertical GRF.

Event	Fatigue	Leg	Fatigue*Leg	Post-hoc		Δ (95% CI)
				ND	D	
IP1	**.** **003**	.083	.106	** **	** **	−0.21 (−0.34, −0.08)
AP	.197	.**030**	.262	** **	** **	** **
IP2	.268	.963	.514	** **	** **	** **

*P*-values associated to the factors *Fatigue* (Pre, Post), *Leg* (Dominant(D), Non-dominant (ND)) and their interactions resulting from the repeated measure ANOVA at different timepoints. Mean differences (Δ) between the pre- and post-values of the vGRF are reported, along with the corresponding 95% confidence intervals (CI). Significant *p*-values are highlighted in bold.

#### 3.3.2 Hip joint moments

The ANOVA indicated no significant differences between the hip moments in the sagittal plane before and after the fatiguing protocol for any leg. However, the effect of *Fatigue* was close to significant at both IP1 and Peak, suggesting a trend towards a reduced joint extension moment. In the frontal plane, we found a significant Fatigue*Leg interaction at IP1, with post-hoc analysis revealing a significant reduction in hip adduction moment for the dominant leg. In the transverse plane, we found no significant differences due to *Fatigue* for any leg. Results of the statistical analyses are reported in Table 5.

**Table 5:** Statistical analyses for hip joint moments.

Plane	Event	Fatigue	Leg	Fatigue*Leg	Post-hoc		Δ (95% CI)
					ND	D	
*Sagittal*	IP	.091	.057	.772	** **	** **	** **
	Peak	.077	.073	.625	** **	** **	** **
*Frontal*	IP	.760	.742	.**016**	.132	**.042**	D: −0.46 (−0.2, −0.04)
	Peak	.170	.**032**	.385	** **	** **	** **
*Transverse*	IP	.513	.592	.134	** **	** **	** **
	Peak	.188	.555	.527	** **	** **	** **

*P*-values associated to the factors *Fatigue* (Pre, Post), *Leg* (Dominant(D), Non-dominant (ND)) and their interactions resulting from the repeated measure ANOVA at different timepoints, as well as *p*-values associated to the post-hoc tests to evaluate potential differential effects of fatigue across legs. Mean differences (Δ) between the pre- and post-values of the joint angle are reported, along with the corresponding 95% confidence intervals (CI). Significant *p*-values are highlighted in bold. IP: impact peak at landing 1; Peak: peak joint moment during landing 1.

#### 3.3.3 Knee moments

In the sagittal plane, there was a significant effect of *Fatigue* independently of *Leg* both at IP1 and at the timepoints of the peak values, with a reduced extension moment. In the frontal plane we found no significant differences due to *Fatigue*. Finally, in the transverse plane there was a significant Fatigue*Leg interaction at the timepoint of peak moment, with post-hoc tests indicating a reduced intra-rotation peak moment in the dominant leg. Results of the statistical analyses are reported in Table 6.

**Table 6:** Statistical analyses for knee joint moments.

Plane	Event	Fatigue	Leg	Fatigue*Leg	Post-hoc		Δ (95% CI)
					ND	D	
*Sagittal*	IP	**<.001**	.986	.841	** **	** **	−0.33 (−0.5, −0.16)
	peak	.**012**	.646	.806	** **	** **	−0.18 (−0.31, −0.04)
*Frontal*	IP	.899	.**043**	.884	** **	** **	** **
	peak	.179	.**006**	.651	** **	** **	** **
*Transverse*	IP	.672	.793	.103	** **	** **	** **
	peak	.064	.994	.**020**	.89	**.002**	D: 0.75 (0.27, 1.22)

*P*-values associated to the factors *Fatigue* (Pre, Post), *Leg* (Dominant(D), Non-dominant (ND)) and their interactions resulting from the repeated measure ANOVA at different timepoints, as well as *p*-values associated to the post-hoc tests to evaluate potential differential effects of fatigue across legs. Mean differences (Δ) between the pre- and post-values of the joint angle are reported, along with the corresponding 95% confidence intervals (CI). Significant *p*-values are highlighted in bold. IP: impact peak at landing 1; Peak: peak joint moment during landing 1.

#### 3.3.4 Ankle moments

There were no significant alterations in ankle moments due to *Fatigue* in any plane. Detailed results of the statistical analyses are reported in Table 7.

**Table 7:** Statistical analyses for ankle joint moments.

Plane	Event	Fatigue	Leg	Fatigue*Leg	Post-hoc		Δ (95% CI)
					ND	D	
*Sagittal*	IP	.502	.095	.833	** **	** **	** **
	peak	.204	.268	.667	** **	** **	** **
*Frontal*	IP	.106	.249	.276	** **	** **	** **
	peak	.281	.649	.161	** **	** **	** **

*P*-values associated to the factors *Fatigue* (Pre, Post), *Leg* (Dominant(D), Non-dominant (ND)) and their interactions resulting from the repeated measure ANOVA at different timepoints, as well as *p*-values associated to the post-hoc tests to evaluate potential differential effects of fatigue across legs. IP: impact peak at landing 1; Peak: peak joint moment during landing 1.

## 4 Discussion

The purpose of this study was to evaluate whether NMF increased the risk of ACL injury. To this end, we developed a fatiguing protocol that took into account the fitness level of each participant. Such a protocol assured us to expose subjects to a normalized anaerobic exercise, inducing an intense acute workload that may increase injury risk (53). We investigated the effects of such a subject-tailored fatiguing exercise on SLDJ kinematics and kinetics, evaluating whether it caused movement alterations that have been identified as risk factors for anterior cruciate ligament (ACL) injury (27, 29). The analyses revealed a reduction in jumping performance following the fatiguing exercise, with several kinematics alterations in the direction of an increased risk of ACL injury (Figures 2A,B), including the “knee dynamic valgus” (29). On the other hand, we observed a reduction or no change in kinetic parameters associated with joint loading, potentially indicating that the CNS adopts protective strategies in the fatigued condition (Figure 2C).

### 4.1 Fatigue-induced detrimental effects on jumping performance

SLDJ performance was assessed by jump height(the maximum height reached by the pelvis during the jump minus the height of the pelvis during quite standing). We observed a reduction in jump height following the fatiguing exercise, but only in the non-dominant leg. We calculated contact time during the first landing as an index of the capability of participants to quickly express force and to efficiently use elastic muscle-tendon properties (54). Interestingly, contact time was not altered by NMF, similarly to what Howard et al. observed (42). While it is possible that these aspects influencing contact time are less affected by NMF than force production (which affect jump height), we should also note that participants—despite being young and active and having familiarized with SLDJ execution—had different sport backgrounds. This could have influenced SLDJ technique and increased variability, potentially masking milder effects of NMF.

### 4.2 Alterations in movement kinematics following NMF

Following NMF we observed a significant alteration in landing strategy, with both knee and hip joints in a more extended position at foot contact. Similarly, the hip joint was more extended during the absorption phase right after the first landing (KF1). These results suggest that participants in the fatigued condition increased or anticipated knee extensor muscles activation prior to landing, adopting a quadriceps-dominant strategy associated to increased ACL strain (27, 32). Electromyographic analyses (32, 55) would need to be performed to confirm this idea. However, similar kinematics alterations have been observed in healthy subjects following cycling exercise (13), during continuous shuttle-run tests at the time of change of directions (56), in patients long after ACL injury treatments (57), as well as in subjects who prospectively developed an ALC rupture (29), suggesting that our fatiguing protocol indeed induced movement alterations associated to ACL injury. The fact that the observed changes in joint kinematics happen especially at foot contact and during the first phases of the SLDJ movement, suggests that there was a recalibration of motor planning (58) following NMF. The disruptive effect of NMF on movement planning has been recently suggested by McLean and Samorzeov, where they observed contralateral effects of localized NMF on movement execution of anticipated and unanticipated single-leg landings (35). These effects may originate from altered mechanisms of sensorimotor integration of proprioceptive information from the fatigued muscles (59), as observed in studies of motor control during volitional movements (14).

On the sagittal plane, we observed significant alterations in the direction of the knee valgus, with an increased abduction of the tibia with respect to the femur during the absorption phase (KF1). This mechanism is consistently associated with the risk of ACL injury (29, 30). Similarly, we found an increased abduction of the hip after fatigue. While previous work associated the occurrence of ACL injuries to increased hip adduction (29, 60, 61), those studies evaluated the biomechanics of bilateral drop landing, differently from the single-leg movements investigated here. In bilateral landing, an inward collapse of the knee (causing an increase in knee abduction) is typically associated with increased hip adduction due to the mechanical constraints between the ground and both legs, forming a closed kinematic chain. On the other hand, in single-leg landing an inward collapse of the knee may need to be compensated by an ipsilateral tilting of the pelvis and the trunk, avoiding a medial displacement of the center of mass that may cause a loose of balance due to the single support. This ipsilateral pelvis and trunk tilt may result in the increased hip abduction observed here. Consistently, previous research showed higher hip abduction angles in single-leg compared to bilateral landings (61). It is worth noting that this mechanism may increase the lever arm of the ground reaction force on the knee joint in the frontal plane, potentially leading to higher external knee abduction moments (or higher internal knee adduction moments) that increase the risk of ACL injury (29). Consistent with this idea, recent research has shown that in soccer most of the ACL injuries occurs during single-leg support, and involve ipsilateral trunk tilt, hip abduction, knee valgus and externally rotated foot (30). The latter is consistent with the increase in hip and knee external rotation observed here following the execution of the fatiguing protocol.

It may be worth noting that NMF caused relatively small, albeit significant, mean kinematic alterations (about 1–2 degrees; see Tables 1–3) when compared to the mean differences found between ACL injured patients and controls in previous studies (8–10 degrees) (29). Since we tested healthy subjects who did not have injuries during the experiments, it was expected to find smaller pre-post fatigue differences than patients-control differences. In addition, NMF may be a less prominent risk factor than others (27, 34), leading to smaller kinematic differences that, yet, are in the direction of an increased risk. Therefore, whether the NMF-induced alterations we found here are clinically relevant is still unclear. Additional experiments and analyses will need to be performed to tackle this issue.

### 4.3 Protective alterations in movement kinetics following NMF

Considering the observed increase in knee and hip extension at foot contact, interpreting the reduction in vertical GRF upon landing is not trivial. The reduction of vertical GRF impact peak may indicate that the CNS developed protective strategies to reduce joint loading (62–64), potentially mediated by joint (65) and muscle afferents (66). These mechanisms may result in a redistribution of force absorption across joints (37) or in a reduction of joint stiffness (40), which would cause longer force dissipation with reduced force peak. Additional experiments using musculoskeletal modelling will be instrumental to estimate joint loading, hence directly testing these hypotheses. These results are in line with previous studies which found a similar reduction in vGRF following fatiguing tasks (12, 34, 37).

Despite significant differences in joint kinematics and reduced vGRF, we did not find many consistent changes in joint moments following NMF. The reduction of impact peak knee extension moments is consistent with the hypothesized reduction of joint stiffness (with longer force dissipation and, indeed, lower peak values), while the reduction of the active peak knee extension moment is likely related to a reduction of jumping performance due to fatigue. The reduction of hip adduction moment in the dominant leg may be due to the reduced GRF, which would lead to lower internal moments given unchanged lever arms. However, many other joint moments appeared to be unaffected by NMF. In particular, we surprisingly found no significant difference in knee adduction moments, a parameter highly related to ACL injury risk (29). As discussed earlier, it is possible that the higher knee and hip abduction angles post-fatigue caused an increased lever arm of the GRF around the knee in the frontal plane, leading to similar adduction moments despite reduced GRF. This would generally make the knee more susceptible to injuries in uncontrolled environments, where unpredictable situations may cause high GRFs (41, 67, 68). Alternatively, these unchanged joint moments may result from a high variability in landing movements across participants, leading to statistically insignificant results.

In general, subjects may exhibit different motor strategies during the execution of potentially hazardous movements. This motivated single-subject analyses in previous studies, that evaluated the frequency of potentially harmful fatigue-induced alterations within the tested population (56). While applauding those results, we decided to perform a global statistical analysis in an attempt to identify general trends using a subject-tailored fatiguing protocol. Yet, we recognize the importance of single-subject analyses, which may identify athletes particularly susceptible to deleterious effects of neuromuscular fatigue on injury risk, and therefore motivate individualized neuromuscular training strategies (69). We will address this issue in future studies, where a higher number of repetitions will be executed for each subject to allow such a within-subject analysis while controlling for each participant's fitness level.

### 4.4 Differential effects of NMF on dominant and non-dominant leg

Interestingly, we observed differing trends and significant changes both in jumping performance and in joint kinematics depending on the tested leg. The influence of laterality on several movements’ parameters have been extensively studied (70, 71) and the increased unbalanced performance between dominant (D) and non-dominant (ND) limbs (e.g., strength, balance, flexibility) has been linked to an increased injury risk (72). To date, only a few studies have investigated the potentially differential effects of NMF on joint parameters depending on laterality. Our results suggest that jumping performance and joint kinematics parameters of the ND leg have been more impacted by the fatiguing protocol. Two possible yet complimentary explanations of our results are plausible. First, previous research suggested that subjects tend to rely on the ND limb for tasks involving strength or explosive power production, such as jumping (71). Therefore, participants may have not been familiar with jumping and landing with the dominant leg, leading to less optimized movements with a high across-subject variability that may have resulted in non-significant results. We limited this factor by allowing participants to familiarize themselves with the execution of SLDJ movements prior to testing. However, there is evidence of sport-specific lateralization (70, 71) which might persist even after familiarization. Second, there may have been a more pronounced contribution of the non-dominant leg to the fatiguing cycling exercise, resulting in a more-persistent post-exercise impairment.

We induced fatigue using a cycle ergometer, a high-intensity exercise that physiologically induced both general (i.e., cardiopulmonary) and local (i.e., metabolites accumulation) effects (73). The resulting NMF clearly encompasses neurophysiological alterations at both the peripheral (i.e., muscles) and central (i.e., brain structures) level of the neuromuscular system (74, 75). Considering this complex physiological interplay, it is not trivial to determine which NMF component (i.e., central or peripheral) most affects our results. Additional experiments, isolating or limiting the NMF-related afferent feedback to the CNS, could address this issue. Potentially, these experiments will determine whether the kinematic alterations observed here are due to the effects of NMF on sensorimotor integration, affecting planning and control strategies during landing (causing additional extension upon landing) and propulsion (causing additional knee extension and abduction).

### 4.5 Limitations

We sampled participants from the general population of healthy, young adults, without addressing a specific population of athletes. This choice reflects the idea of using a subject-tailored fatiguing protocol, which should elicit comparable fatigue-induced physiological responses across a potentially heterogeneous set of participants. However, the heterogeneity of the tested participants may have introduced variability in motor performance and movement biomechanics at baseline (i.e., before the execution of the fatiguing protocol). The use of more restrictive inclusion criteria could have reduced inter-subject variability and provided more insightful, yet more restrictively applicable, information on sport-specific populations.

The height of the step used in the present study was equal for all the participants. Despite none of the participants felt uncomfortable during the movement and step height was comparable to those used in similar studies (13, 29, 61, 76), the impact of step height on movement biomechanics may vary depending on participants’ height. While this aspect may have introduced variability in the data, we believe it has a limited impact on our results, as participants’ heights and BMIs were relatively similar to one other. In addition, the use of a pre-post design (along with the corresponding repeated measures statistical tests used here), reduces the influence of inter-subject variability, hence limiting the potential impact of employing a single step height or that originating from the diverse sport backgrounds across participants. Yet, it is still possible that some non-significant results reported here, such as the unaltered contact time following fatigue, may originate from these considerations.

In this study, we used a constant workload cycling exercise tailored to each participant's fitness level. However, in many sports (e.g., football), intermittent instead of constant-load exercise is frequently performed. Future studies will need to be performed to design an intermittent fatiguing protocol, tailored to participant's fitness level. This new study will allow us to re-evaluate the results presented here in a more ecological and sport-specific context. Finally, additional experiments will need to be performed to analyze alterations in muscle activity following a subject-tailored fatiguing protocol, evaluating whether NMF causes altered muscle patterns that could increase ACL strain.

## Data Availability

The raw data supporting the conclusions of this article will be made available by the authors, without undue reservation.
